# Correction: Gibbs process distinguishes survival and reveals contact-inhibition genes in Glioblastoma multiforme

**DOI:** 10.1371/journal.pone.0315099

**Published:** 2024-12-03

**Authors:** Afrooz Jahedi, Gayatri Kumar, Lavanya Kannan, Tarjani Agrawal, Jason Huse, Krishna Bhat, Kasthuri Kannan

The fourth author’s name is spelled incorrectly. The correct name is: Tarjani Agrawal. The correct citation is: Jahedi A, Kumar G, Kannan L, Agrawal T, Huse J, Bhat K, et al. (2023) Gibbs process distinguishes survival and reveals contact-inhibition genes in Glioblastoma multiforme. PLOS ONE 18(2): e0277176. https://doi.org/10.1371/journal.pone.0277176.
