# Supplementary figures and images for: Gibbs process distinguishes survival and reveals contact-inhibition genes in Glioblastoma multiforme
Source: PLoS One. 2023 Feb 16;18(2):e0277176. doi: 10.1371/journal.pone.0277176 (PMC9934342; doi:10.1371/journal.pone.0277176)

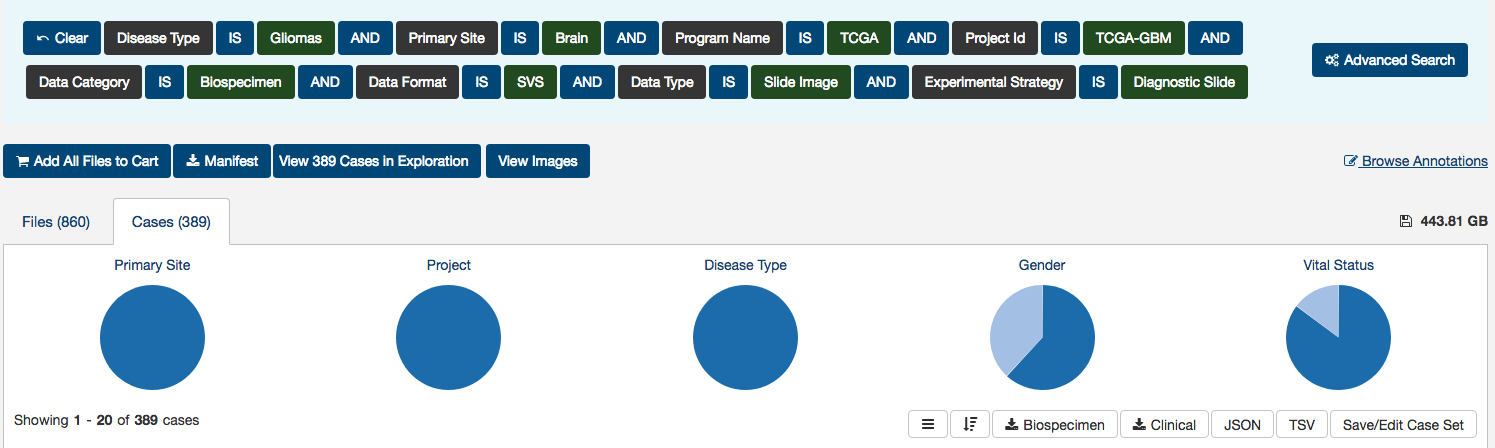

Supplement: S1 File — (PNG) [file pone.0277176.s001.png]

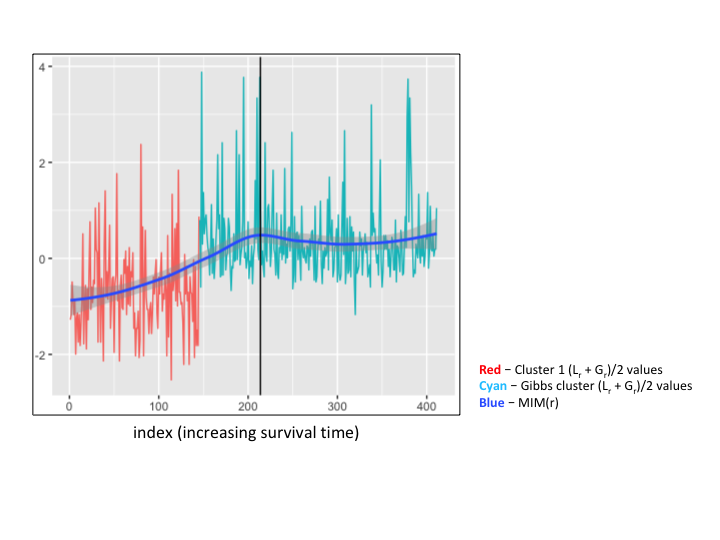

Supplement: S1 Fig — (PNG) [file pone.0277176.s018.png]

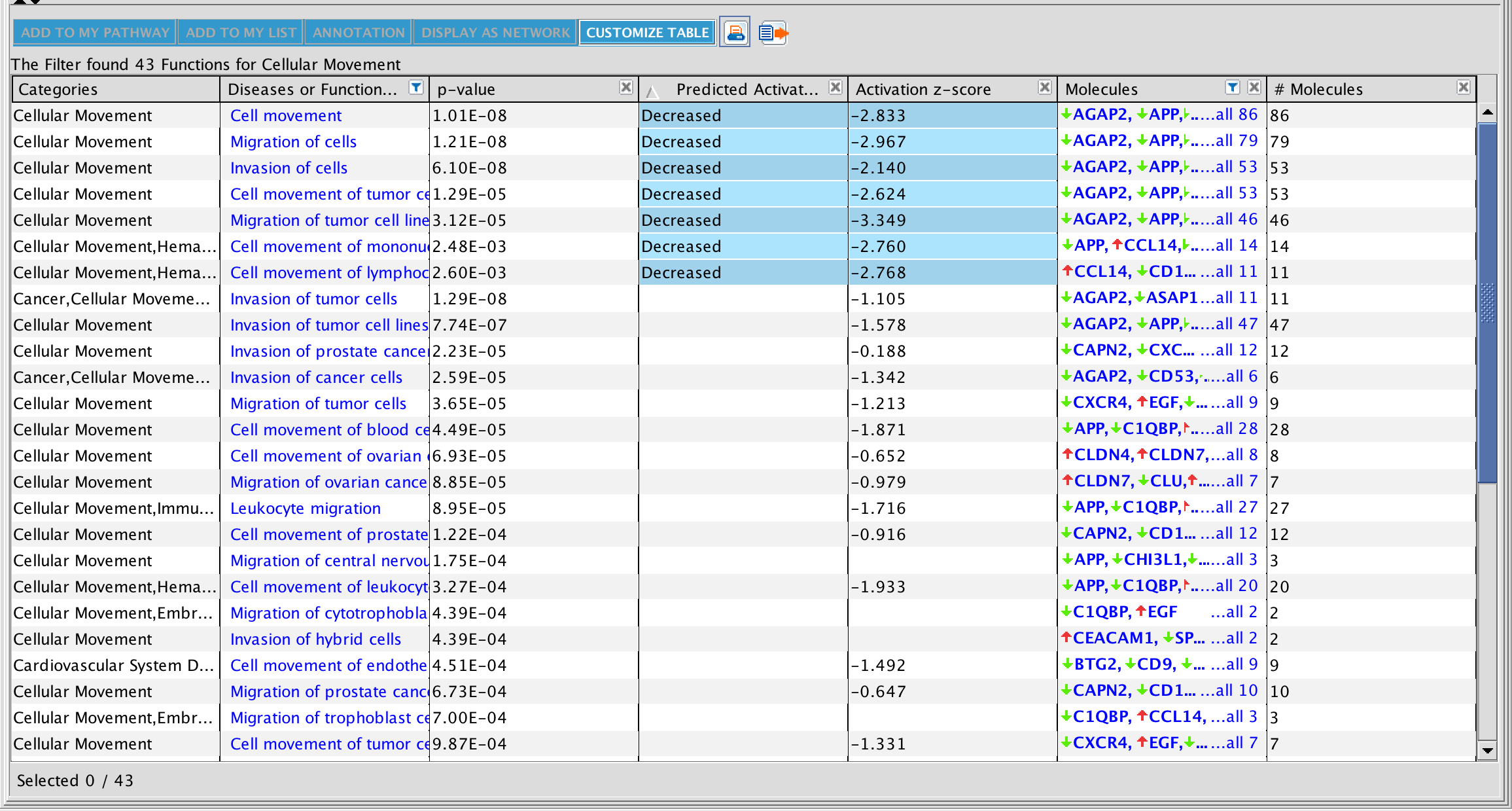

Supplement: S2 Fig — (PNG) [file pone.0277176.s019.png]

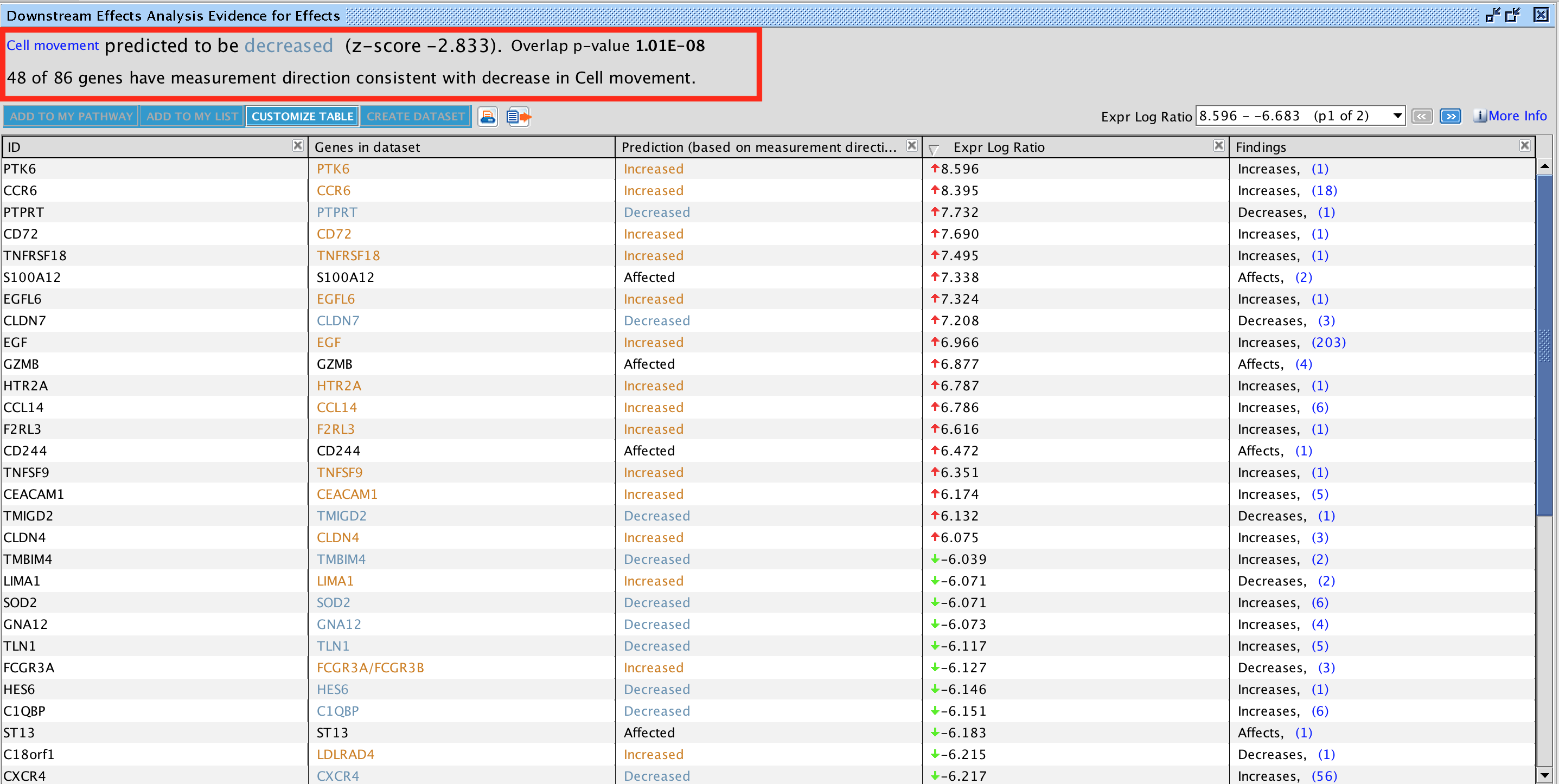

Supplement: S3 Fig — (PNG) [file pone.0277176.s020.png]

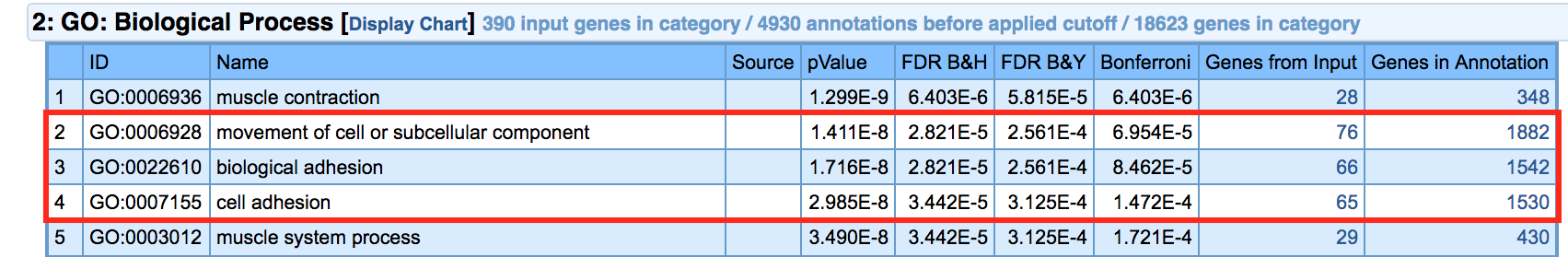

Supplement: S4 Fig — (PNG) [file pone.0277176.s021.png]

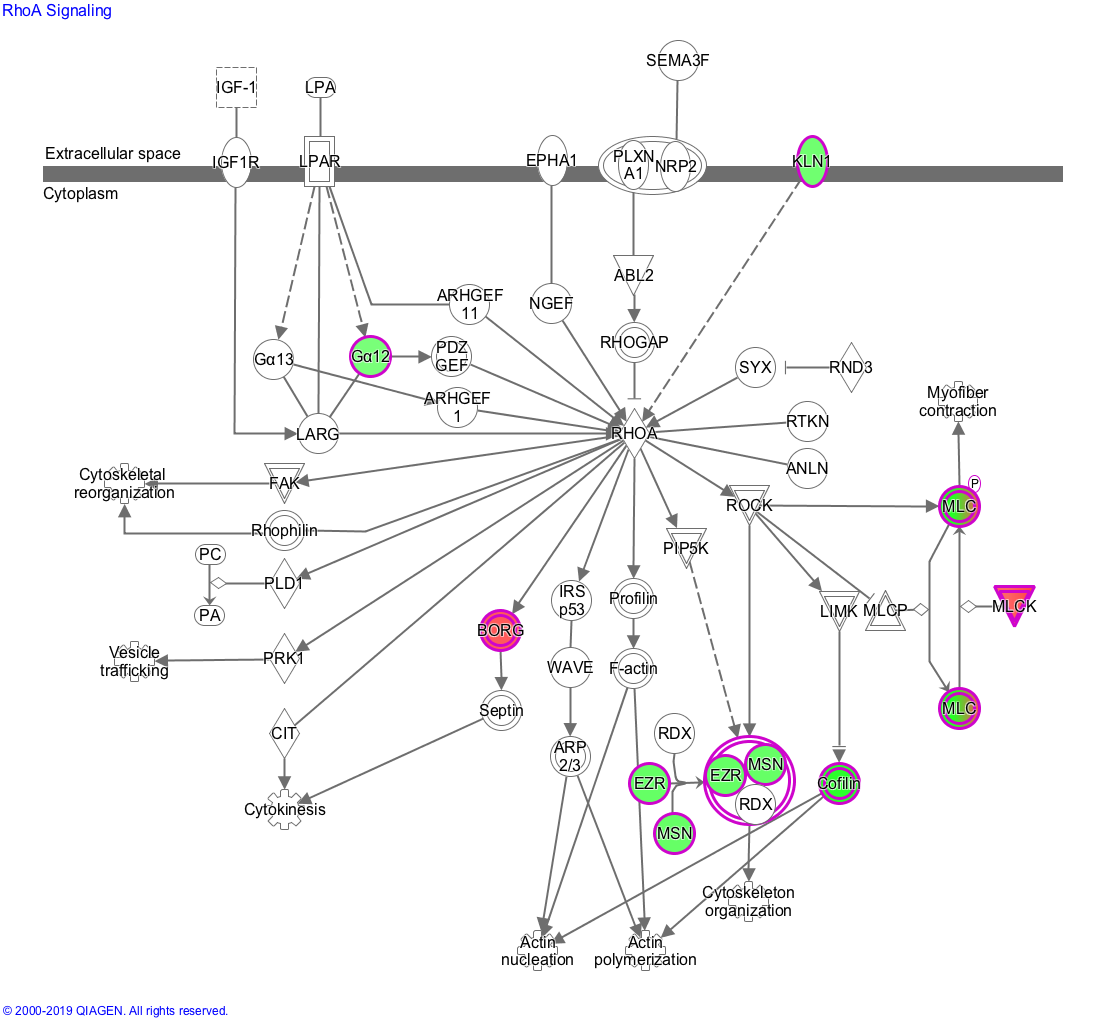

Supplement: S5 Fig — (PNG) [file pone.0277176.s022.png]

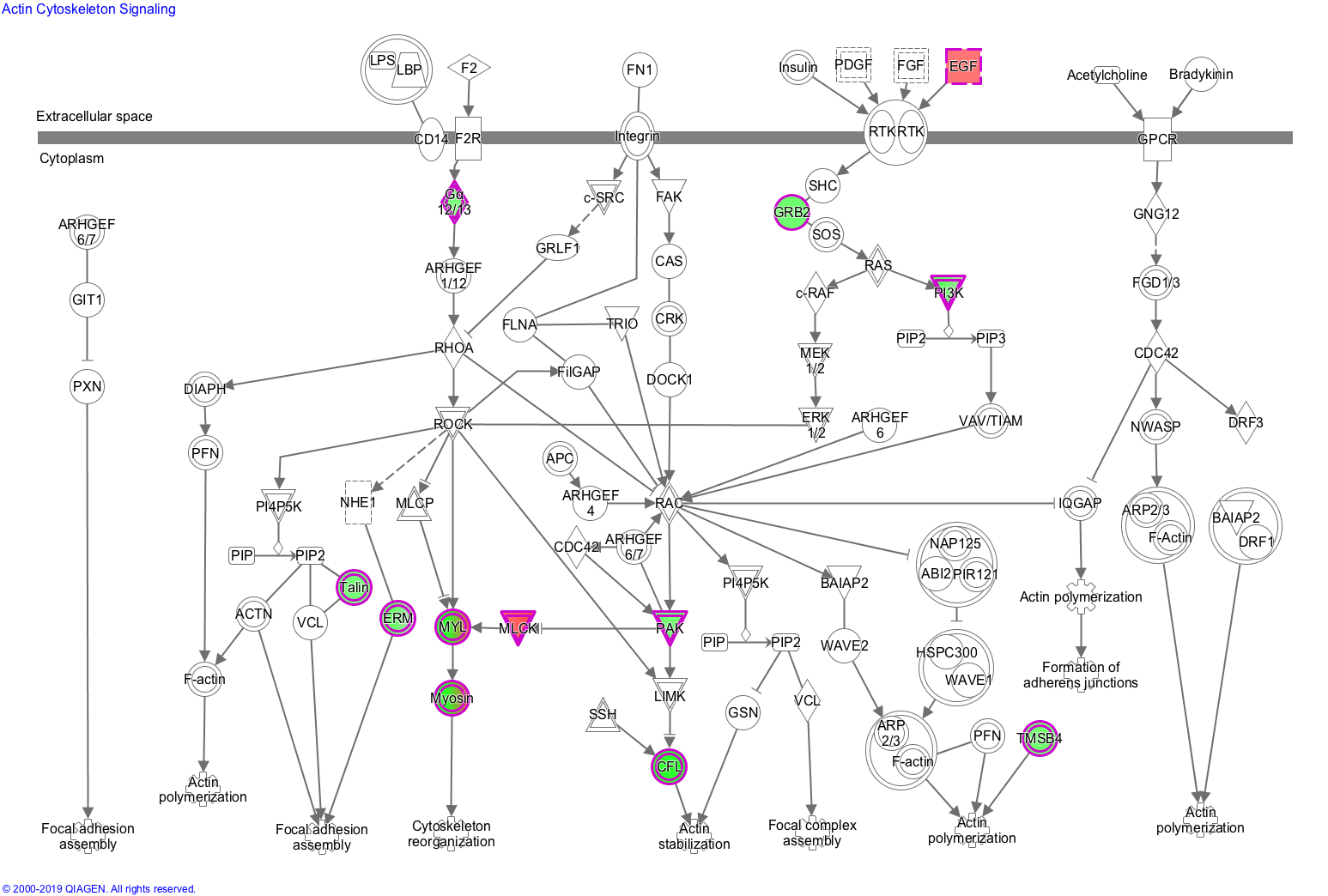

Supplement: S6 Fig — (PNG) [file pone.0277176.s023.png]

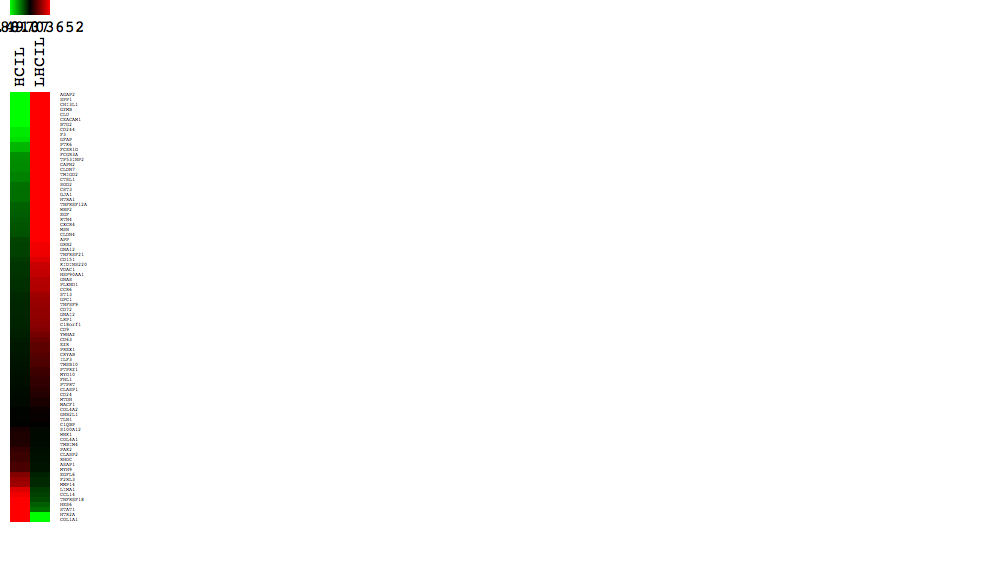

Supplement: S7 Fig — (TIFF) [file pone.0277176.s024.tiff]
